# Effects of Gold Nanoparticles on *Mentha spicata* L., Soil Microbiota, and Human Health Risks: Impact of Exposure Routes

**DOI:** 10.3390/nano14110955

**Published:** 2024-05-29

**Authors:** Alexandra Peshkova, Inga Zinicovscaia, Liliana Cepoi, Ludmila Rudi, Tatiana Chiriac, Nikita Yushin, Tran Tuan Anh, Ho Manh Dung, Serghei Corcimaru

**Affiliations:** 1 Joint Institute for Nuclear Research, 6 Joliot-Curie Str., 141980 Dubna, Russia; peshkova.alexandra92@gmail.com (A.P.); ynik_62@mail.ru (N.Y.); 2 Doctoral School Biological, Geonomic, Chemical and Technological Science, State University of Moldova, 60 Alexei Mateevici Str., MD-2009 Chisinau, Moldova; 3 Horia Hulubei National Institute for R&D in Physics and Nuclear Engineering, 30 Reactorului Str., 077125 Măgurele, Romania; 4 Institute of Microbiology and Biotechnology, Technical University of Moldova, 1 Academiei Str., MD-2028 Chisinau, Moldova; liliana.cepoi@imb.utm.md (L.C.); ludmila.rudi@imb.utm.md (L.R.); tatiana.chiriac@imb.utm.md (T.C.); serghei.corcimaru@imb.utm.md (S.C.); 5 Department of Nuclear and Isotopic Technique, Dalat Nuclear Research Institute, 01 Nguyen Tu Luc, Dalat 670000, Vietnam; ttanhfr@yahoo.com (T.T.A.); dungmanhho@gmail.com (H.M.D.)

**Keywords:** gold nanoparticles, foliar spraying, root irrigation, spearmint, k0-based neutron activation analysis, biochemistry, herbal infusion

## Abstract

Nanoparticles, due to their extensive production and application, can have significant consequences for the environment, including soil and plant pollution. Therefore, it is very important to assess how nanoparticles will affect plants depending on the exposure pathways. The effect of gold nanoparticles in a concentration range of 1–100 mg/L on *Mentha spicata* L. during a 28-day experiment was investigated. Two routes of nanoparticles exposure were applied: root and foliar. Transmission electron microscopy was used to characterize nanoparticles and their effect on plant leaves’ ultrastructure. Gold content in soil and plant segments was determined using k0-neutron activation analysis. For root exposure, gold was mainly accumulated in soil (15.2–1769 mg/kg) followed by root systems (2.99–454 mg/kg). The maximum accumulation of gold in leaves (5.49 mg/kg) was attained at a nanoparticle concentration of 100 mg/L. Foliar exposure resulted in the maximum uptake of gold in leaves (552 mg/kg) and stems (18.4 mg/kg) at the highest applied nanoparticle concentration. The effect of nanoparticles on the *Mentha spicata* L. leaves’ biochemical composition was assessed. Nanoparticles affected the content of chlorophyll and carotenoids and led to an increase in antioxidant activity. Root exposure to gold nanoparticles resulted in an increase in the number of starch grains in chloroplasts and also suppressed the activity of the soil microbiota. Gold extraction from mint leaves into herbal infusion varied from 2 to 90% depending on the concentration of nanoparticles in the solution and the exposure route. The health risk as a result of gold exposure via herbal tea intake was assessed through estimated daily intake. The hazard quotient values were found to be less than the cutoff, indicating that a cup of tea infusion should not cause a serious impact to human health.

## 1. Introduction

The extensive growth of nanomaterial production is a result of wide application of engineered nanoparticles in medicine and the pharmaceutical industry, the agricultural sector, electronics, water treatment, etc. Besides industrial application, nanoparticles are present in various consumer products, including cosmetics, suntan lotions, paints, and stain-resistant clothing [1,2,3,4,5]. Thus, gold and silver nanoparticles are used as components of lotions, antiperspirants, and anti-aging face creams, and they help to eliminate lesions and improve skin condition. The size of gold nanoparticles (AuNPs) used in skin care products usually ranges from 5 to 400 nm [6,7]. It should be noted that AuNPs play an important role in medicine, where they act as carriers of drugs, amplifiers and optical signal converters, and are used in the diagnosis and treatment of cancer tumors [8,9,10,11]. Due to the increased production of engineered nanoparticles and their use in various fields, their release into natural and artificial ecosystems becomes inevitable [12]. 

AuNPs are often introduced into agricultural ecosystems—both accidentally and intentionally. In recent years, nanoparticles have been actively used as fertilizers and pesticides since it was proven that particles with sizes less than 100 nm have higher biological activity as compared to larger materials—due to their higher surface-area-to-volume ratio [13,14]. It was suggested that nanofertilizers or nanoencapsulated nutrients have beneficial effects on crops. Thus, to combat plant diseases and to increase plant productivity, nanotechnological techniques for targeted particle delivery have been developed. In experiments with *Cucurbita pepo*, it was demonstrated that iron–carbon nanoparticles applied thought injections and spraying were able to penetrate and migrate in the plant body [13]. Field spraying of *Brassica juncea* leaves with AuNPs positively influenced various parameters related to plant growth and yield, including plant height, stem diameter, and number of branches and pods [14]. Another study reported an increase in the rate of seed germination and growth of *Gloriosa superba* after treatment with AuNPs at a concentration of 1000 μM [15]. The use of AuNPs as fertilizer improved the synthesis of ginsenosides in ginseng and enhanced the anti-inflammatory effects of red ginseng [16]. In *Arabidopsis thaliana*, AuNPs in concentrations up to 80 µg/L enhanced total seed yield and improved seed germination rate, vegetative growth, and free-radical-scavenging activity [17]. 

At the same time, extensive use of engineered nanomaterials results in their release in the environment and accumulation in soil [18], which in turn leads to their uptake by plants, resulting in potentially negative impacts on human health [19,20,21]. A number of studies showed that nanoparticles present in the atmosphere can settle on leaves, penetrate them through trichomes or stomata, and then be transferred to other plant tissues [22]. Luo and Cao [20] found that the plants *Erigeron canadensis* and *Boehmeria nivea* collected in the Guangdong Province of China contained AuNPs of various shapes with a diameter of 5–50 nm [20].

Since gold is not one of the biologically important trace elements, its accumulation in plants, besides beneficial effects, can lead to toxic impacts as well [12]. Thus, the length of *Arabidopsis thaliana* L. roots when grown in the presence of 100 mg/L AuNPs was 75% shorter [23]. AuNPs can also affect the soil microbial community. Maliszewska [24] reported that AuNPs showed toxicity towards soil microorganisms involved in carbon and nitrogen transformations. Nanoparticles can be accumulated in plants through roots and leaves, and while the accumulation through roots is relatively well-studied, their uptake through leaves is less investigated and requires additional research. Accumulation of nanoparticles in plants consumed as food by the human population may result in their transfer into the human body. To our knowledge, there is no research on the complex impact of metal nanoparticles on soil microbiota, plants, and human health. 

The aim of the present study was to assess the effect of AuNPs applied in different concentrations through root and foliar application on *Mentha spicata* L. The objectives of this study included the following: (i) assessment of gold accumulation in soil and different segments of plants using neutron activation analysis; (ii) investigation of the AuNPs effect on soil microbiota and Mentha biochemical composition; (iii) calculation of the percentage of gold extraction in herbal infusion; and (iv) evaluation of the potential health risks of spearmint infusions containing AuNPs.

## 2. Materials and Methods

### 2.1. Object of Study—Mentha spicata *L.*

As the object of this study, spearmint (*Mentha spicata* L.), a perennial herbaceous plant of the genus Mentha, family *Lamiaceae*, was selected. The choice of Mentha is due to its wide cultivation area and active use in the food, medical, and cosmetics industries. The aerial parts of spearmint, which have antioxidant, anticancer, and antimicrobial effects, are used to obtain essential oil and flavor cosmetics and food products. Leaves, stems, and flowers are traditionally used for brewing tea and infusions used as a tonic for fatigue, headaches, toothaches, and diabetes, as a diuretic, and in the case of intestinal problems [25,26,27,28].

### 2.2. Nanoparticles

Gold nanoparticles coated with polyvinylpyrrolidone were purchased from M9 (Tolyatti, Russia). For the experiments, solutions with concentrations of 1, 5, 10, 50, and 100 mg/L were prepared by diluting the initial concentrate (200 mg/L) with deionized water obtained using the Adrona water purification system.

### 2.3. Soil

For the experiments, the universal nutrient soil S-2 (VELTORF LLC, Velikiye Luki, Russia), the basis of which is a mixture of peat of varying degrees of decomposition, was selected. The soil also contains ammonium nitrate phosphate fertilizer, limestone meal, 100–180 mg/L of NH_4_^+^NO_3_, 100–180 mg/L of P_2_O_5_, and 130–260 mg/L of K_2_O. The acidity of the aqueous soil suspension is 5.5. 

### 2.4. Experimental Design

The cuttings of spearmint were planted in 200 mL pots filled with soil and for two weeks were watered with tap water. Once the plants reached a height of 20 cm, their treatment (root or foliar) with AuNPs in concentrations of 1, 5, 10, 50, and 100 mg/L started. Plants were treated once every two days for 28 days. The plants were cultivated with a 12 h light/12 h dark photoperiod, at a temperature of 20–21 °C and a humidity of 20–25%. The volume of solution applied for root irrigation was 20 mL, and when spraying foliage—2 mL. The selected volumes of solutions are considered optimal from the point of view of the plant’s moisture needs, preventing dripping during leaf spraying and percolation through the soil when irrigating. Spraying of nanoparticle solutions onto the surface of foliage was carried out using a polypropylene aerosol with preventive soil screening. Control plants were grown under similar laboratory conditions; however, tap water was used for irrigation and spraying. At the end of the experiment, soil samples were taken from each pot, and the plants were washed and then divided into segments (root, stem, and leaf) for elemental and biochemical analyses and transmission electron microscopy (TEM). For elemental analysis, samples were dried at 50 °C for 72 h, homogenized, and further dried at 105 °C for 24 h. Fresh mint leaves were frozen and stored at −80 °C for biochemical analyses; leaf preparation for TEM will be presented later. 

### 2.5. Soil Microbial Activity

Soil microbial activity was estimated by measuring the CO_2_ emission rate (soil respiration). A total of 25 g of air-dried control soil (no treatment with AuNPs) and soil treated with AuNPs at a concentration of 100 mg/L were placed into 250 mL Erlenmeyer flasks, re-moistened by adding 9 mL of distilled water, and pre-incubated for 30 days in the dark at 25 °C. Each sample was prepared in three replicas. Prior to the estimation, the soil samples had been kept in an air-dry state for 1 month.

The CO_2_ efflux in each flask was measured by using the closed dynamic chamber methodology with the help of an Li-850 infrared gas analyzer (LI-COR, Lincoln, NE, USA). The rate of increase in the CO_2_ concentration in the chamber air, which included the air in the flask, tubing, and the infrared gas analyzer, was measured. The obtained data were converted into the emission rate expressed in μg of C-CO_2_ from 1 g of soil per hour. The CO_2_ emission rate was calculated according to the following formula [29]:

R_s_ = 3600 × DCO_2_ × M_C_ × P/P_i_ × V/V_i_ × T_i_/T/W_s_ (1)

where R_s_ is the CO_2_ emission rate, 3600 = the number of seconds in 1 h, ΔCO_2_ is the change in CO_2_ concentration (micromoles of CO_2_ in 1 mole of air) per second in the chamber air, M_C_ = 12.01 g (the molar mass of C), P = pressure (kPa), P_i_ = 101.325 k Pa (standard pressure), V = air volume (L) in the chamber, V_i_ = 22.41 L (volume of a mole of air at 0 °C), T_i_ = 273.15 K (standard temperature), T = temperature (K), and W_s_ = mass (g) of the tested soil. 

### 2.6. Analytical Techniques

#### 2.6.1. Transmission Electron Microscopy

For TEM analysis, fresh leaves were cut into squares with dimensions of 1 mm × 1 mm and placed in Eppendorf tubes (total volume 2 mL) containing 1.5 mL of 2.5% glutaraldehyde solution in PBS fixation buffer. PBS buffer composition (g/L): NaCl 8; KCl 0.2; Na_2_HPO_4_, 1.44; NaH_2_PO_4_ 0.2; and pH 7.0. After fixation, the material was washed twice to remove any remaining fixing solution in the PBS buffer. Next, samples were subjected to dehydration using 30% ethanol and were filled with the resin solution. Ultrathin sections were prepared using an LKB AB ultramicrotome (LKB-Produkter, Kiruna, Sweden). TEM images were obtained on the transmission electron microscope (Thermo Scientific Talos F200i, Thermo Scientific, Waltham, MA, USA).

#### 2.6.2. k0-Neutron Activation Analysis

For neutron activation analysis (NAA), which was carried out at the Dalat research reactor (Dalat, Vietnam), samples were dried and homogenized, then packed in polyethylene bags. Soil samples were irradiated for 10 h and plant samples for 15 h at the rotary rack with a thermal neutron flux of 3.8 × 10^12^ n·cm^−2^·s^−1^. After neutron irradiation, the samples were decayed for an appropriate time according to each element group and measured on a gamma spectrometer using a GMX-30190 (ORTEC, Zoetermeer, The Netherlands) with an efficiency of 30%, with the energy resolution at 1332.5 keV of 60Co is 2.1 keV. The gamma spectrum was acquired through the MAESTRO program. 

The samples were co-irradiated with a gold monitor (IRMM 530, Al-0.1% Au) to determine the thermal neutron flux at the irradiation position. The quality control of the results was provided via the irradiation of certified reference materials: NIST-2709a (San Joaquin Soil), NIST-2711a (Montana II Soil), and NIST-1570a (Spinach leaves).

### 2.7. Biochemical Composition of Biomass, Antioxidant Activity, and Malondialdehyde Content

Extraction of chlorophyll from spearmint biomass was performed using 90% acetone. The absorbance of the extract was measured spectrophotometrically at three different wavelengths: 645 nm, 652 nm, and 663 nm. The resulting measurements are used to calculate the total chlorophyll content, expressed in milligrams per 100 g of biomass. To extract carotene, 96% ethanol was used. The absorbance was measured at 450 nm and the results were calculated in mg/100 g of biomass.

The antioxidant activity of the spearmint extract, prepared using a 50% hydro-ethanolic solution, was determined by measuring the reduction in the radical 2,2′-azino-bis (3-ethylbenz-thiazoline-6-sulfonic acid (ABTS) over a period of 6 min. The antioxidant activity was expressed as a % of ABTS inhibition.

To determine the level of lipid peroxidation by quantifying the content of malondialdehyde (MDA), the thiobarbituric acid (TBA) assay was used according to the method proposed by Heath and Packer [30]. For the reaction, 150 mg of plant tissue was macerated and 3 mL of reagent solution containing 0.25% thiobarbituric acid prepared in 10% trichloroacetic acid was added. The homogenate was heated in a water bath at 95 °C for 30 min. After cooling and centrifuging the samples, absorbance was recorded at 532 nm and 600 nm wavelengths. The MDA concentration was calculated using the extinction coefficient.

### 2.8. Assessment of Au Translocation

The bioconcentration factor (*BCF*) was defined as the ratio of element content in plant segments to its content in soil [31]:

(2) $$BCF=C_{R\;(St,\;\;\;L)}/C_{S\;}$$

The translocation coefficient is the ratio of element content in the stem or leaves to its content in the roots as well as the ratio of element content in the leaves to its content in the stem:

(3) $$TF=C_{St\;or\;L}/C_{R\;or\;St}$$

where *C_R_* is the gold content in the roots, *C_St_* is the gold content in the stems, *C_L_* is the gold content in the leaves, and *C_S_* is the gold content in the soil (all values in mg/kg).

When spraying the leaf surface, the coefficient of translocation from the above-ground part to the root system was calculated as the ratio (3).

### 2.9. Human Health Risk Assessment

The estimated daily intake (EDI) of gold was calculated according to Equation (4)

(4) $$EDI=\frac{C_{i}\times IR\times EF\times ED}{AT\times BW\times 1000}$$

where *C_i_* is gold concertation in the infusion, μg/L; *IR* is the tea ingestion rate, 1250 mL per person per day; *EF* is the exposure frequency, 365 days/year; *ED* is the exposure duration, 70 years; *AT* is the average exposure time, *EF* × *ED*; and *BW* is the average body weight, 70 kg [32]. 

The hazard quotient (*HQ*) of gold in tea infusion was calculated to qualitatively assess the non-carcinogenic effects of metal on humans through tea consumption (Equation (5)):

(5) $$HQ=\frac{EDI}{RfD}$$

where *RfD* is daily oral reference dose, μg·kg/day. Due to lack of relevant toxicological data, a value of 1.32 μg·kg/day was used in the calculation. This value was calculated based on the consumption of products containing E175 (gold containing food additive) [33].

## 3. Results and Discussion

### 3.1. Nanoparticles Characterization

**Figure 1:**
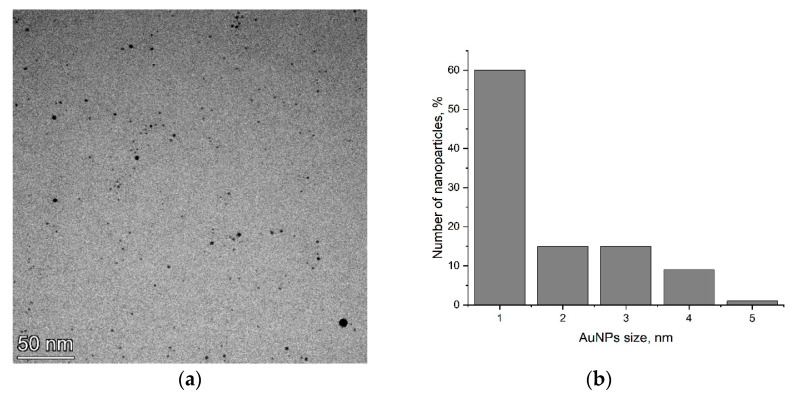
TEM image of AuNPs (**a**) and their size distribution (**b**).

TEM was applied to determine the size of the AuNPs. According to Figure 1, AuNPs with a size of 1–5 nm are uniformly distributed in solution and do not form aggregates. 

### 3.2. Gold Content in Soil and Nanoparticles’ Effect on Soil Microbiota

**Figure 2:**
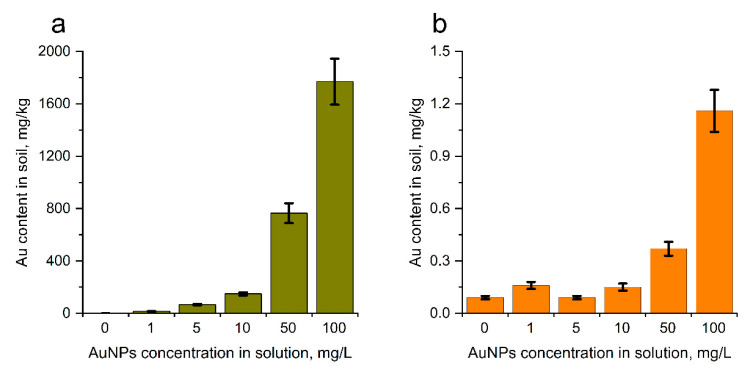
The content of gold in soil samples under root irrigation condition (**a**) and foliar spraying with AuNPs (**b**), determined with k0-NAA (AuNPs concentrations 1–100 mg/L, duration of experiment 28 days).

The content of gold in the control soil used for experiments was 0.09 mg/kg. Watering of soil with AuNPs solutions of different concentrations resulted in the direct increase in gold content in soil. Thus, at AuNPs concentration of 1 mg/L it constituted 15.2 mg/kg, while at the highest concentration of 100 mg/L, levels reached 1769 mg/kg (Figure 2a). Previously, Malejko et al. [34] showed that AuNPs were accumulated in roots, stems, and leaves of potato plants, with a much higher concentration remaining in the soil. The same finding was reported by [35].

*Mentha spicata* L. treatment via spraying did not influence gold content in soil at AuNPs concentrations of 1–10 mg/L. At AuNP concentrations of 50 and 100 mg/L, gold content in the soil was 4- and 13-times higher than in control, respectively (Figure 2b). In [36], it was found that under foliar application of 5–15% of AuNPs, AuNPs with a diameter less than 50 nm were released into the rhizosphere soil, and the main part of nanoparticles were accumulated in young shoots and roots. When comparing two procedures of plant treatment with AuNPs, it was observed that with watering, gold content in soils was 95–2067 times higher than when spraying was applied. 

**Figure 3:**
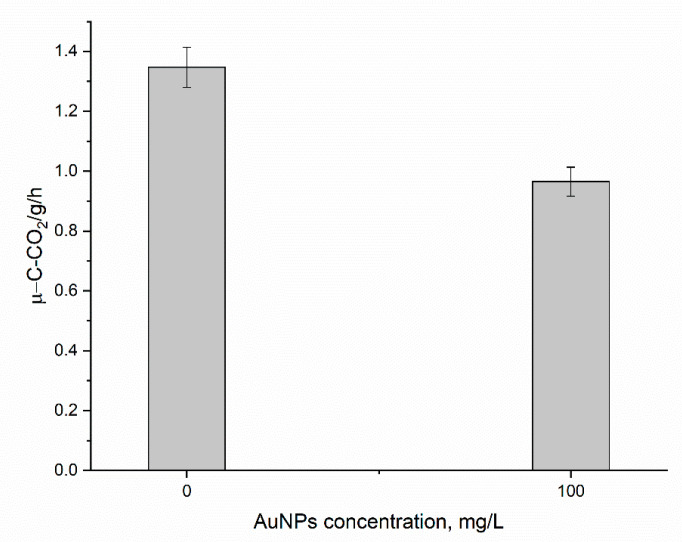
Soil respiration rates in the variants with root irrigation of AuNPs in the concentrations of 0 (Control) and 100 mg/L (AuNPs). The error bars represent the confidence intervals at *p* = 0.05.

Soil is the largest receptor for the NPs and their effect on soil depends on NPs concentration, soil type, and enzymatic activity of soil [37]. Investigating the effect of NPs on the activity of dehydrogenase, urease, acidic, and alkaline phosphatase in two soils [38] showed that, depending on the enzyme and soil type tested, an inhibitory or a stimulating effect of NPs on the activity of the enzymes can be observed. In the present study, the soil respiration rate in the sample watered with AuNPs was 28% lower than in the control (Figure 3). This inhibition effect on soil microbial activity was statistically significant and implied potential environmental risks resulting from AuNPs’ accumulation in soil. Further research into the nature and extent of the impact of AuNPs’ accumulation on soil microbial activity is necessary to prevent long-term negative consequences of soil pollution by AuNPs on soil health.

### 3.3. Gold Content in Mentha Spicata Segments

**Figure 4:**
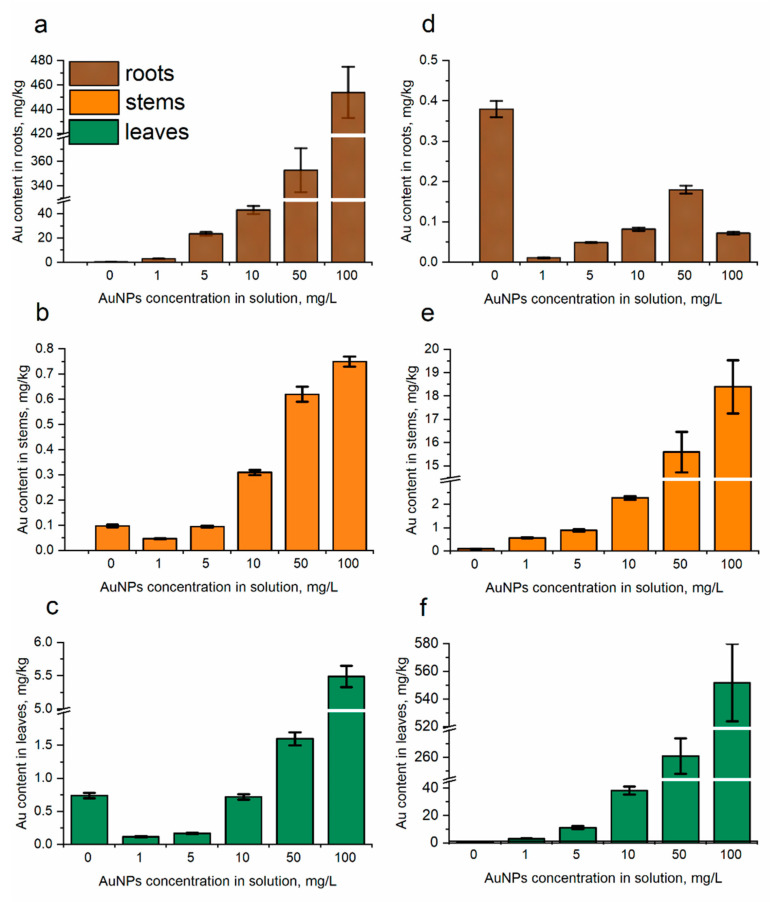
The content of gold in spearmint’s segments: (**a**–**c**) under root irrigation condition and (**d**–**f**) foliar spraying with AuNPs determined via k0-NAA (NPs concentrations 1–100 mg/L, duration of experiment 28 days).

Roots, stems, and leaves of control plants contained 0.38, 0.09, and 0.74 mg/kg of gold, respectively. Under conditions of mint watering with AuNPs, a directly proportional dependence of the gold content in the soil, roots, and leaves was observed (Pearson’s correlation coefficient (r) at *p* < 0.005 was 0.99, 0.97, and 0.97, respectively). The highest accumulation of gold was in roots (2.99–454 mg/kg) followed by leaves (0.12–5.49 mg/kg) and stems (0.05–0.52 mg/kg) (Figure 4). Additionally, if in roots gold content increased compared to control at all applied AuNP concentrations, in steam and leaves an increase was observed only at AuNP concentrations of 50 and 100 mg/L.

An important aspect in the uptake and transport of metal-containing nanoparticles is their availability to plants, which is associated with various parameters such as soil properties, pH, temperature, physicochemical properties of nanoparticles and type of coating [20,34]. Plants are able to absorb gold in soluble forms and transport it to the aboveground parts; however, in a reducing environment, precipitation occurs on the cell surface, inhibiting membrane permeability [39]. Gao et al. [40] reported high AuNP extraction from soil in the pH range of 4.0–10, while Reith and Cornelis [41] showed their high stability in soil. 

Sabo-Attwood et al. [42] demonstrated that citrate-coated AuNPs with a size of 3.5 nm easily penetrated *Nicotiana xanthi* from a hydroponic solution, while no absorption was recorded for particles with a size of 18 nm. A number of studies have reported that AuNPs can be taken up by plant roots and transported through plant tissues, but the localization in plant segments may vary [34,43,44]. Zhu et al. [45] showed that positively charged AuNPs were mainly accumulated in plant roots, while negatively charged particles were translocated into stems and leaves. The same authors demonstrated that plants accumulated AuNPs differently; thus, in radish and ryegrass, AuNPs were mainly accumulated in roots, while in rice and pumpkin they are mainly accumulated in shoots. Exposure of *Petroselinum crispum* to solutions of AuNPs in a concentration range of 1–200 mg/L resulted in their high uptake in roots but also in active transfer in leaves [35].

Spraying of mint plants with AuNPs resulted in the significant increase in gold content in leaves and stems (at *p* < 0.005 r = 0.99 and 0.95, respectively). The content of gold in leaves increased from 3.2 to 552 mg/kg and was 4–746-times higher than the control values. In stems, gold content varied from 0.57 to 18.4 mg/kg and was 6–188-times higher compared to control (Figure 4b). Obtained results are in good agreement with the data reported earlier for *Brassica juncea* [14]. Plant spraying with solutions of AuNPs in concentrations of 0, 10, 25, 50, and 100 ppm resulted in an increase in gold content in leaf tissues with increasing concentration of the applied AuNPs. It should be mentioned that, in case of foliar application of AuNPs, there was no accumulation of gold in roots. However, gold nanostructures of 30–80 nm firstly accumulated in watermelon leaves and were then translocated to the roots via the phloem transport mechanism [46]. The foliar uptake of nanoparticles occurs through stomatal and cuticular entrances [47]. Avellan et al. [36], studying the effect of AuNPs with different coatings on their accumulation in wheat leaves, showed that citrate-coated nanoparticles are accumulated more actively than PVP-coated AuNPs during spraying. The authors suggest that the mechanism of uptake of PVP-coated AuNPs occurs in part through disruption and diffusion of nanoparticles through the cuticle layer and also possibly through stomata. In *Perilla frutescens*, AuNPs were accumulated through stomatal pathways [47].

### 3.4. The Effect of Plant Treatment with AuNPs on the Ultrastructure of Leaves 

**Figure 5:**
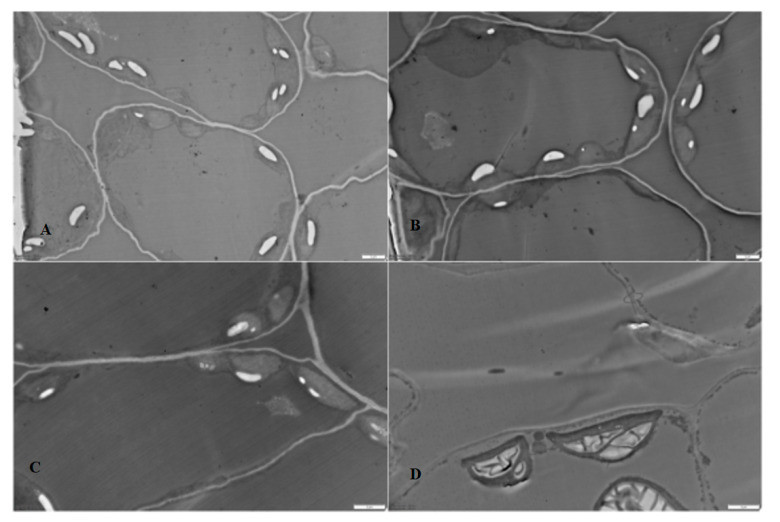
Transmission electron micrographs of spearmint leaf cells: control (**A**,**B**); under foliar spraying with AuNPs (**C**); and under root irrigation with AuNPs (**D**), scale bar—2 µm.

The ultrastructure of leaves of plants treated with AuNP was analyzed using TEM (Figure 5). 

In control and both variants involving the treatment of plants with AuNP solutions, leaf cells are characterized by a typical structure with the presence of a massive tonoplast and numerous chloroplasts, within which starch granules are clearly observed. The latter are present both in the chloroplasts of control plants’ leaves and in those of plants treated with nanoparticle solutions. Differences are observed in the case of leaves of plants irrigated with gold nanoparticle solutions. In this case, chloroplasts are practically entirely filled with starch granules.

### 3.5. Treated Plants’ Biochemical Analysis and Antioxidant Activity

**Figure 6:**
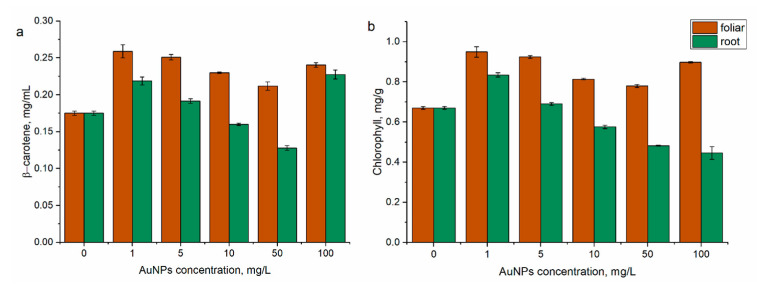
Change in the content of (**a**) β-carotene and (**b**) chlorophyll under the action of AuNPs (root and foliar treatment).

Mint plants sprayed with AuNPs exhibited an increase in β-carotene content in the leaves at almost all applied concentrations (Figure 6a). At AuNP concentrations of 1 and 5 mg/L β-carotene content increased by 48% (*p* < 0.05) and 43.5% (*p* < 0.001), respectively, compared to the control sample. High concentrations of AuNP, of 50 and 100 mg/L, induced an increase in pigment content by 21% (*p* < 0.05) and 37.4% (*p* < 0.05), respectively. In the case of plants sprayed with 10 mg/L of AuNPs, β-carotene content was reduced by 32% (*p* < 0.05) in comparison with control.

For mint plants watered with AuNPs, the highest values of β-carotene content were obtained at nanoparticle concentrations of 1 mg/L (by 24.9%, *p* < 0.05) and 100 mg/L (by 29.9%, *p* < 0.05). The AuNP concentration of 10 mg/L reduced the pigment content 8.6% (*p* < 0.05) compared to control, while the lowest values were recorded at a concentration of 50 mg/L (by 26.8%, *p* < 0.001). 

In mint leaves treated with AuNPs via spraying, the chlorophyll content increased by 16.5–41.5% compared to the control (Figure 6b). The highest increase of 41.5% (*p* < 0.05) and 37.7% (*p* < 0.001) was noticed at AuNP concentrations of 1 and 5 mg/L. For mint leaves watered with AuNPs, chlorophyll levels increased by 24.5% (*p* < 0.001) at an AuNP concentration of 1 mg/L, and chlorophyll levels were comparable to the control at a AuNP concentration of 5 mg/L. AuNPs in concentrations of 10–100 mg/L caused a decrease in chlorophyll content by 14.2–33.5%.

Mezacasa et al. [48] showed that AuNPs interact with chlorophyll and can change the operation of the plants’ photosynthetic system. In *Adsotech’s jatamansi*, plants grown in the presence of AuNPs exhibit an increase in the level of chlorophyll a and b, carotenoids, and total chlorophyll [49]. At the same time, treatment of wheat with AuNPs led to an increase in the chlorophyll content at a nanoparticle concentration of 10 mg/L and it was lower than in the control at higher concentrations [50]. 

**Figure 7:**
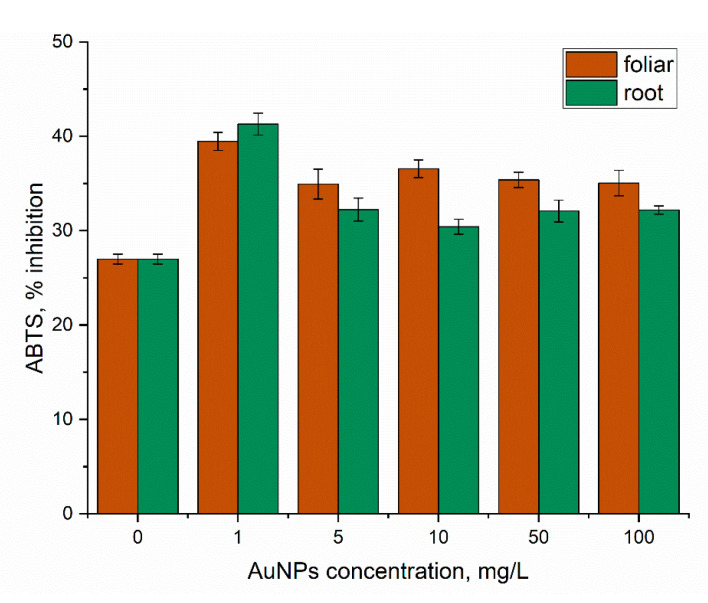
Modification of the antioxidant activity of ethanolic extracts obtained from mint leaves under the action of AuNPs (root and foliar treatment).

Treatment of mint with AuNPs through spraying and watering resulted in the increase in antioxidant activity by 46.2% (*p* < 0.001) and 52.9% (*p* < 0.001), respectively (Figure 7). 

Spraying of mint plants with AuNPs in the concentration range of 5–100 mg/L led to an increase in antioxidant activity of 39.4–35.4%. Watering of plants with nanoparticles showed a more modest increase in the antioxidant activity values, by 12.7–19.4% compared to control. AuNPs increased the antioxidant activity of *Nardostachys jatamansi* plants treated [49].

**Figure 8:**
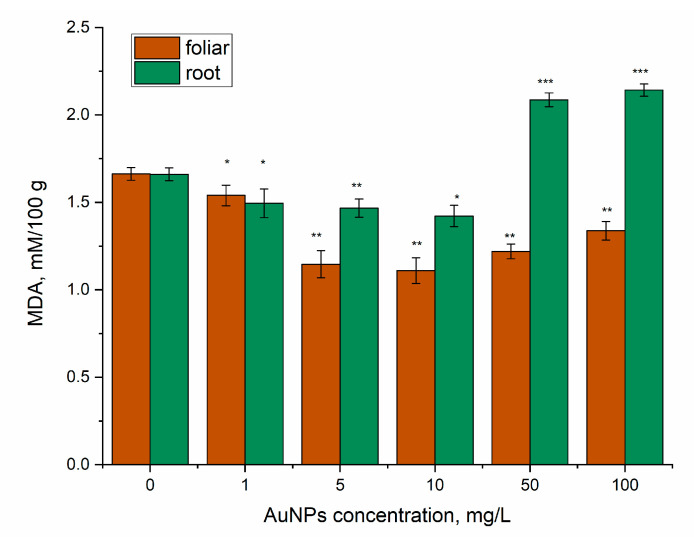
The content of malondialdehyde in mint leaves under action of AuNPs (root and foliar exposure). * *p* < 0.05; ** *p* < 0.005; *** *p* < 0.001.

Besides the antioxidant activity, the content of MDA in mint leaves also changed; the difference being dependent on the route of AuNP administration (Figure 8). Thus, for AuNP application via spraying, a statistically significant decrease in the amount of MDA of up to 33.2% compared to the control was obtained. When plants were irrigated with AuNPs in concentrations of 1, 5, and 10 mg/L, the content of malonic dialdehyde in plants decreased compared to the control (by 10–13%), while application of AuNP in concentrations of 50 and 100 mg/L resulted in an increase in MDA content by 25.7% and 29.0%, respectively, compared to the control. MDA is a well-known marker of oxidative stress, and the increase in its content indicates oxidative stress. In the present study, comparing the MDA test results with other obtained data (such as the increase in β-carotene content and the decrease in chlorophyll content), it can be assumed that concentrations of 50 and 100 mg/L of AuNPs applied through irrigation can cause oxidative changes in mint, including potential degradation of biologically active compounds in plant biomass, consequently decreasing its therapeutic value. 

### 3.6. Translocation Factor (TF) and the Bioconcentration Factor (BCF)

The values of TF and BCF calculated for Mentha spicata are presented in Table 1. 

In the conditions of root treatment with AuNPs, the BCF values for all plant segments were below the cutoff. The same pattern was seen for TF, except leaves/stems, where TFs were higher than the cutoff. In the case of plants that were sprayed, all TFs were <1.0. This is in agreement with findings reported by Feichtmeier et al. [51] and Judy et al. [52]. Aggregation of the nanoparticles leading to a lower actual concentration of gold nanoparticles available for plant uptake may be one of the reasons of their low translocation [51,53]. Zhai and co-authors showed that gold in ionic form was more actively transported from roots to leaves in comparison with gold in the nanoform [54]. Accumulation of AuNPs also depends on plant species. For example, tobacco accumulated gold of different size and with different surface coating, while no accumulation of gold was observed in wheat [52].

### 3.7. Assessment of Gold Transfer from Plants in the Infusion and Health Risk Assessment

Since *Mentha spicata* is actively utilized to prepare tea and infusions, the transfer of gold from plants treated with AuNPs to an infusion was assessed (Table 2). Infusions prepared from control plants contained 0.66 mg/L of gold, and the extraction rate was on the level of 2%. The concentration of gold in the infusions prepared from mint subjected to root treatment with AuNPs varied from 0.124 to 81.76 µg/L. A high correlation was observed between gold content in leaves and its concentrations in infusions (r = 0.99 at *p* < 0.005). In total, 2–45% of gold was extracted into the infusions. The concentrations of gold in leaves treated with AuNPs through spraying varied between 0.04 and 18 mg/L and was in direct ratio to gold content in mint leaves (r = 0.98 at *p* < 0.005). The extraction of gold in infusions was on the level of 2–90%.

The estimated daily intake (EDI) values of gold through the consumption of tea infusion and hazard quotient values are summarized in Table 3. The EDI values for infusions prepared from the leaves subjected to foliar spraying with AuNPs were 38–300-times higher compared to plants watered with AuNPs. The higher the concentration of gold in the infusion, the large the EDI values. The TQ values, independent of the method of AuNP exposure, for individuals as a result of the ingestion of infusions were all below the cutoff, suggesting that the daily intake of gold via the consumption of such infusions would be unlikely to result in adverse health effects, if we consider daily intake as a benchmark at the level of 1.32 µg/kg [33]. 

It should be noted that there is no established reference dose for gold, as it is considered that due to the low solubility of elemental gold, its bioavailability is low, resulting in minimal health effects [55]. However, it has been demonstrated that even at very low doses, orally administered AuNPs are accumulated in significant amounts in various organs, including reproductive ones, and their presence can be detected even after a clearance period. Additionally, AuNPs possess high biological activity and may cause long-term or delayed effects [56].

## 4. Conclusions

In the present study the effect of gold nanoparticles applied through root and foliar routes on *Mentha spicata* was investigated. Neutron activation allowed for the assessment of differences in gold accumulation in plant segments under different methods of exposure. For root exposure, gold content in the soil was directly proportional to nanoparticle concentrations in solutions, while in roots and leaves it increased only at AuNP concentrations of 50 and 100 mg/L. Plants sprayed with solutions of AuNPs resulted exhibited high accumulation of gold in leaves and stems, which attained values of 552 mg/kg and 18.4 mg/kg, respectively, at nanoparticles concentration of 100 mg/L. For root irrigation, the activity of soil microbiota decreased by 28% compared to control. The content of pigments in *Mentha spicata* leaves increased when spraying with solutions of AuNPs and decreased for plants watered with high concentration of NPs. An increase in the antioxidant activity was observed in both cases and the level of the malonic dialdehyde, an oxidative stress marker, increased significantly for plants irrigated using high concentrations of NPs. High extraction of gold from plants into infusions indicates an increased risk of element trophic transfer, while suppression of soil microbiota activity points at potential risks of nanoparticle accumulation for the environment. Although EDI and HQ values, calculated based on a daily intake of E175 of 1.32 ug/kg, indicate that there is no risk of adverse health effects provoked by the consumption of infusions from leaves containing AuNPs, long-time exposure experiments need to be conducted to better understand the effect of gold nanoparticles on human health. 

## Figures and Tables

**Table 1:** Bioconcentration factor (BCF) and translocation factor (TF) values for spearmint treated with AuNPs (root and foliar treatment).

Concentration of AuNPs in Solution, mg/L	BCF			TF		
	Roots	Stems	Leaves	Stems/Roots	Leaves/Stems	Leaves/Roots
Root irrigation						
1	0.197	0.003	0.008	0.016	2.553	0.040
5	0.353	0.001	0.003	0.004	1.789	0.007
10	0.290	0.002	0.005	0.007	2.323	0.017
50	0.461	0.001	0.002	0.002	2.581	0.005
100	0.257	0.0004	0.003	0.002	7.320	0.012
Foliar spraying						
				Roots/Stems	Stems/Leaves	Roots/Leaves
1				0.019	0.176	0.003
5				0.055	0.077	0.004
10				0.036	0.059	0.002
50				0.012	0.060	0.001
100				0.004	0.033	0.0001

**Table 2:** Gold extraction from leaves of mint treated with AuNPs into infusion.

Concentration of AuNPs in Solution	Gold Content in Infusion, mg/L		Gold Content in Leaves mg/kg		Extraction into Infusion, %	
	Root Exposure	Foliar Spraying Condition	Root Exposure	Foliar Spraying Condition	Root Exposure	Foliar Spraying Condition
0	0.66	0.66	0.74	0.74	2	2
1	0.12	35	0.12	3.2	2	22
5	3.8	148	0.17	11.6	45	26
10	7.3	625	0.72	38.4	20	33
50	16.3	11,760	1.6	261	20	90
100	81	18,726	5.4	552	30	68

**Table 3:** Estimated daily intakes (EDI) and hazard quotient (HQ) of gold due to the consumption of infusion obtained from the mint leaves subjected to treatment with AuNPs.

Gold Content in Infusion, mg/L		EDI		HQ	
Root Exposure	Foliar Spraying Condition	Root Exposure	Foliar Spraying Condition	Root Exposure	Foliar Spraying Condition
0.66	0.66	1.18 × 10^−5^	1.18 × 10^−5^	8.93 × 10^−6^	8.93 × 10^−6^
0.12	35	2.14 × 10^−6^	6.25 × 10^−4^	1.62 × 10^−6^	4.73 × 10^−4^
3.8	148	6.79 × 10^−5^	2.64 × 10^−3^	5.14 × 10^−5^	2.00 × 10^−3^
7.3	625	1.30 × 10^−4^	1.12 × 10^−2^	9.88 × 10^−5^	8.46 × 10^−3^
16.3	11,760	2.91 × 10^−4^	2.10 × 10^−1^	2.21 × 10^−4^	1.59 × 10^−1^
81	18,726	1.45 × 10^−3^	3.34 × 10^−1^	1.10 × 10^−3^	2.53 × 10^−1^

## Data Availability

Data are contained within the article.
